# Effects of phenylbutazone, firocoxib, and dipyrone on the diuretic response to furosemide in horses

**DOI:** 10.1111/jvim.16914

**Published:** 2023-10-26

**Authors:** Julianne M. White, Aimee C. Colbath, Harold C. Schott

**Affiliations:** ^1^ Department of Large Animal Clinical Sciences, College of Veterinary Medicine Michigan State University East Lansing Michigan USA

**Keywords:** cyclooxygenase, diuresis, equine, natriuresis, NSAID

## Abstract

**Background:**

Treatment with phenylbutazone (nonselective COX inhibitor) decreases the diuretic and natriuretic effects of furosemide by nearly 30% but the effects of COX‐2 specific inhibitors (firocoxib) and atypical NSAIDs (dipyrone) are unknown.

**Hypothesis:**

Furosemide‐induced diuresis after pretreatment with firocoxib or dipyrone is diminished to a lesser extent than after pretreatment with phenylbutazone.

**Animals:**

Eight healthy mares.

**Methods:**

Each mare received 4 treatments in a prospective experimental crossover study using a replicated 4 × 4 Latin Square design: furosemide alone (FU), furosemide and phenylbutazone (PB), furosemide and firocoxib (FX), and furosemide and dipyrone (DP). After 24 hours of NSAID treatment at recommended dosages, ureteral catheters were placed for continual urine collection. After a 30‐minute baseline collection period, furosemide (1.0 mg/kg, IV) was administered, and urine and blood samples were collected for 4 hours. Data were assessed by repeated measures ANOVA.

**Results:**

Four‐hour urine volume was (mean ± SD) ~25% less (*P* < .001) after pretreatment with all NSAIDs (PB 19.1 ± 2.1 mL/kg, FX 17.7 ± 3.5 mL/kg, DP 19.1 ± 3.9 mL/kg), as compared to FU (23.4 ± 5.1 mL/kg) (*P* < .001), but there were no differences between PB, FX, or DP. Interindividual variability in furosemide diuresis after pretreatment with different NSAIDs was observed.

**Conclusions and Clinical Importance:**

Though COX‐2 selective NSAIDs and dipyrone might have less severe or fever gastrointestinal adverse effects in horses, our data suggest minimal differences in effects on furosemide‐induced diuresis, and possibly, risk of nephrotoxicosis.

**Abbreviations**
AKI: acute kidney injury
ANOVA: analysis of variance
AVP: arginine vasopressin
COX: cyclooxygenase
COXIB: COX‐2 inhibitor
Cr: serum creatinine concentration
ECF: extracellular fluid
FCl_Cl_
: fractional chloride clearance
FCl_K_
: fractional potassium clearance
FCl_Na_
: fractional sodium clearance
GFR: glomerular filtration rate
GIT: gastrointestinal tract
HR: heart rate
ICF: intracellular fluid
MD: macula densa
NKCC‐2: Na^+^/K^+^/2Cl^−^ cotransporter
PCV: packed cell volume
PG: prostaglandin
RBF: renal blood flow
TAL: thick ascending limb
TS: total solids
USG: urine specific gravity

## 1 INTRODUCTION

Nonsteroidal anti‐inflammatory drugs (NSAIDs) are administered to horses to alleviate pain, fever, and inflammation; however, excessive or prolonged administration can have adverse effects on the gastrointestinal tract (GIT) and kidneys.^1^ NSAIDs inhibit cyclooxygenase (COX) enzymes that catalyze oxidation of arachidonic acid to prostaglandins (PGs). There are several COX isoforms and COX‐1 derived PGs are traditionally viewed as homeostatic, while COX‐2 is considered an “inducible” isoform upregulated with inflammation.^2^

In the kidney, however, both COX‐1 and COX‐2 have “constitutive” expression and activity.^3^ COX‐1 is the predominant isoform in glomerular mesangial cells, arteriolar endothelial cells, and collecting duct epithelium in most species, while COX‐2 is found in glomerular cells, medullary interstitial cells, macula densa (MD) cells, and tubular epithelium in the thick ascending limb (TAL).^3^ COX‐1 derived PGE_2_ attenuates afferent arteriole vasoconstriction in response to decreased renal perfusion and antagonizes the antidiuretic effect of arginine vasopressin (AVP) in collecting ducts, and medullary COX‐2 PGE_2_ production is increased to maintain perfusion of the normally hypoxic medulla during periods of renal hypoperfusion.^3^, ^4^ The overall effect of renal COX activity is to assist with maintenance of renal blood flow (RBF), glomerular filtration rate (GFR), and urine output during normal physiologic conditions and to preserve RBF, especially to the inner medulla, and GFR during renal hypoperfusion.^2^, ^3^

Furosemide is a loop diuretic that causes saluresis, primarily by blocking the apical Na^+^/K^+^/2Cl^−^ cotransporter (NKCC‐2) in the TAL of the loop of Henle.^5^, ^6^ In addition, furosemide activates COX isoforms in different nephron segments. For example, furosemide‐induced COX‐1 PGE_2_ production antagonizes the effect of AVP to increase water reabsorption in collecting ducts^4^ while furosemide‐induced COX‐2 PGE_2_ production in MD cells can lead to further inhibition of tubular reabsorption of Na^+^ and Cl^−^ in the TAL.^7^

Nonselective NSAIDs phenylbutazone and meloxicam inhibit both COX‐1 and COX‐2, and pretreatment with phenylbutazone attenuates the diuretic response to furosemide by ~30%.^8^, ^9^, ^10^ Treatment of horses with meloxicam, a COX‐2 preferential NSAID that becomes nonselective at higher plasma concentrations, produces a similar inhibition of furosemide‐induced diuresis.^10^ Firocoxib, a second generation NSAID highly selective for COX‐2 (a COXIB), was approved for use in horses about a decade ago with a goal of providing a similar analgesic effect as phenylbutazone with less risk of adverse GIT effects.^11^ However, use of COXIBs does not appear to confer “renoprotection” in humans.^12^, ^13^ COXIBs demonstrated attenuation of furosemide diuresis, comparable with nonselective COX inhibitors in cats and rats.^14^, ^15^ Dipyrone, termed an atypical NSAID because of both PG dependent (through COX‐3 activity) and independent effects, is approved as an antipyretic agent for use in horses.^16^ However, use of dipyrone in the intensive care unit has been associated with a dose‐dependent increased risk of acute kidney injury (AKI) in people.^17^ The effects of firocoxib and dipyrone on the renal responses to furosemide in horses are unknown. The purpose of this study was to evaluate furosemide‐induced diuresis after pretreatment with firocoxib or dipyrone, as compared to pretreatment with phenylbutazone. We hypothesized that there would be less inhibition of furosemide‐induced diuresis and natriuresis after pretreatment with firocoxib or dipyrone, as compared to phenylbutazone.

## 2 MATERIALS AND METHODS

### 2.1 Study design

Eight mares were studied 4 times in a replicated 4 × 4 Latin Square design. Treatments included furosemide alone (FU), phenylbutazone and furosemide (PB), firocoxib and furosemide (FX), and dipyrone and furosemide (DP). Randomization was performed using a random pair generator (https://commentpicker.com/combination-generator.php) to determine the order of treatments for each mare. Treatments were randomized independent of studying mares as pairs. Mares were group housed in paddocks and fed grass hay but no concentrate feed or supplements. All procedures in this study were approved by the Michigan State University Institutional Animal Care and Use Committee (PROTO202000077) and animals were cared for according to the principles outlined in the NIH Guide for the Care and Use of Laboratory Animals.

### 2.2 Instrumentation

Two days before furosemide administration, a pair of mares was transported to the hospital where they were housed in individual stalls and fed grass hay and water ad libitum. The next morning (day 1), mares were weighed and an IV catheter (JorVet Extended Use Catheter 14G, Jorgensen Laboratories, Loveland, CO) was aseptically inserted into a jugular vein; left jugular veins were utilized for the first and third treatment phases, and right jugular veins were utilized for second and fourth treatment phases. Between 8 and 9 am (after IV catheter placement), horses received 1 of 4 treatments: (1) FU, 0.9% NaCl (0.02 mL/kg, IV; Hospira, Lake Forest, IL); (2) PB, phenylbutazone (4.4 mg/kg, IV; VetOne, Boise, ID); (3) FX, firocoxib (0.27 mg/kg, IV = loading dose; Equioxx Injection, Merial/Boehringer Ingelheim Animal Health, Duluth, GA); and (4) DP, dipyrone (30 mg/kg, IV; Zimeta, Dechra Veterinary Products, Overland Park, KS). Treatments or catheter flushing were repeated after 12 and 24 hours: (1) FU, 0.9% NaCl (0.02 mL/kg, IV, q 12 hours); (2) PB, phenylbutazone (4.4 mg/kg, IV, q 12 hours); (3) FX, firocoxib (catheter flush with 0.9% NaCl, 0.02 mL/kg, IV at 12 hours and 0.09 mg/kg firocoxib, IV = maintenance dose at 24 hours); and (4) DP, dipyrone (30 mg/kg, IV, q 12 hours) for a total of 24 hours of NSAID treatment.

After medication administration on the morning of day 2, horses were restrained in stocks without sedation, the bladder was emptied by urethral catheterization (EQUIVET Stallion Urinary Catheter, KRUUSE, Langeskov, Denmark) and ureteral catheters were inserted, as previously described (modified 18 French silicone Foley catheter, Bard Urological, Covington, GA; 8 French polypropylene catheter, Cardinal Health, Dublin, OH).^18^ Patency of ureteral catheters was evident when pulsatile urine flow was observed from the ends of each catheter at regular intervals. The ends of the catheters were secured to the vulva with a suture after local anesthesia and an extension tube was connected for urine collection. Mares remained in stocks during the subsequent 4.5 hours urine collection period with access to hay in a hay net and water was offered from a bucket 90 and 180 minutes after furosemide administration. After completion of the 4.5 hours urine collection period, the bladder was catheterized to ensure it remained empty (no leakage of urine around ureteral catheters into the bladder). A single dose of phenylbutazone (4.4 mg/kg, IV; VetOne, Boise, ID) was administered, then IV and ureteral catheters were removed and a single dose of a trimethoprim‐sulfamethoxazole antibiotic combination was administered (25 mg/kg, PO; Amneal Pharmaceuticals, Bridgewater, NJ). Mares were returned to pasture for a minimum of 1 week between treatment phases.

### 2.3 Blood and urine sample collection

Blood for determination of packed cell volume (PCV), plasma total solids (TS), and serum Na^+^, K^+^, Cl^−^ and creatinine (Cr) concentrations was collected via the jugular catheter and heart rate (HR) was determined by auscultation 15 minutes before and at 15, 30, 45, 60, 90, 120, 180, and 240 minutes after furosemide administration. Blood for determination of PCV and TS was transferred to heparinized microhematocrit tubes, spun, and immediately analyzed by the microhematocrit method and refractometry, respectively, in duplicate. Blood was also placed into clot tubes and after 60 to 90 minutes at room temperature, tubes were centrifuged (1500*g* for 15 minutes) and aliquots of serum were stored at −80°C until analysis. Urine was collected continuously from each ureteral catheter. After a 30 minute baseline urine collection period, furosemide (1 mg/kg, IV; VetOne, Boise, ID) was administered through the IV catheter and volumes of urine collected from each ureteral catheter from 0 to 30, 30 to 60, 60 to 120, and 120 to 240 minutes were recorded. Proportional aliquots of urine (from right and left ureters) were pooled into samples representative of total urine production for each urine collection period and, after centrifugation (1500*g* for 15 minutes), aliquots were frozen at −80°C until analysis. Urine specific gravity (USG) was measured by refractometry on all samples before freezing.

### 2.4 Serum and urine analyses

Serum and urine Na^+^, K^+^, Cl^−^ and Cr concentrations were determined via ion selective electrodes and the Jaffe reaction, respectively (AU680 Clinical Chemistry Analyzer, Beckman Coulter, Inc., Brea, CA). Total urine production and excretion of Cr and electrolytes over the 4 hours after furosemide administration were calculated from urine volumes and electrolyte and Cr concentrations for each collection period (urine samples from the 0 to 30 minutes and 30 to 60 minutes collection periods were combined, in proportion, for measurement of electrolyte and Cr concentrations). Fractional electrolyte clearances were calculated for each collection period

### 2.5 Statistical analysis

Data were assessed for normality by the Kolmogorov‐Smirnov test. Data in the text and tables are presented as mean ± SD. Data in the figures are presented as mean ± SEM. Total urine production and excretion of Cr and electrolytes were assessed by a 1‐way repeated measures ANOVA. PCV, TS, and serum electrolyte and creatinine concentrations, along with USG, urine electrolyte concentrations, and fractional clearance of electrolytes were analyzed by a 2‐way repeated measures ANOVA to determine effects of time and treatment. Significance was set at *P* < .05.

## 3 RESULTS

**FIGURE 1:**
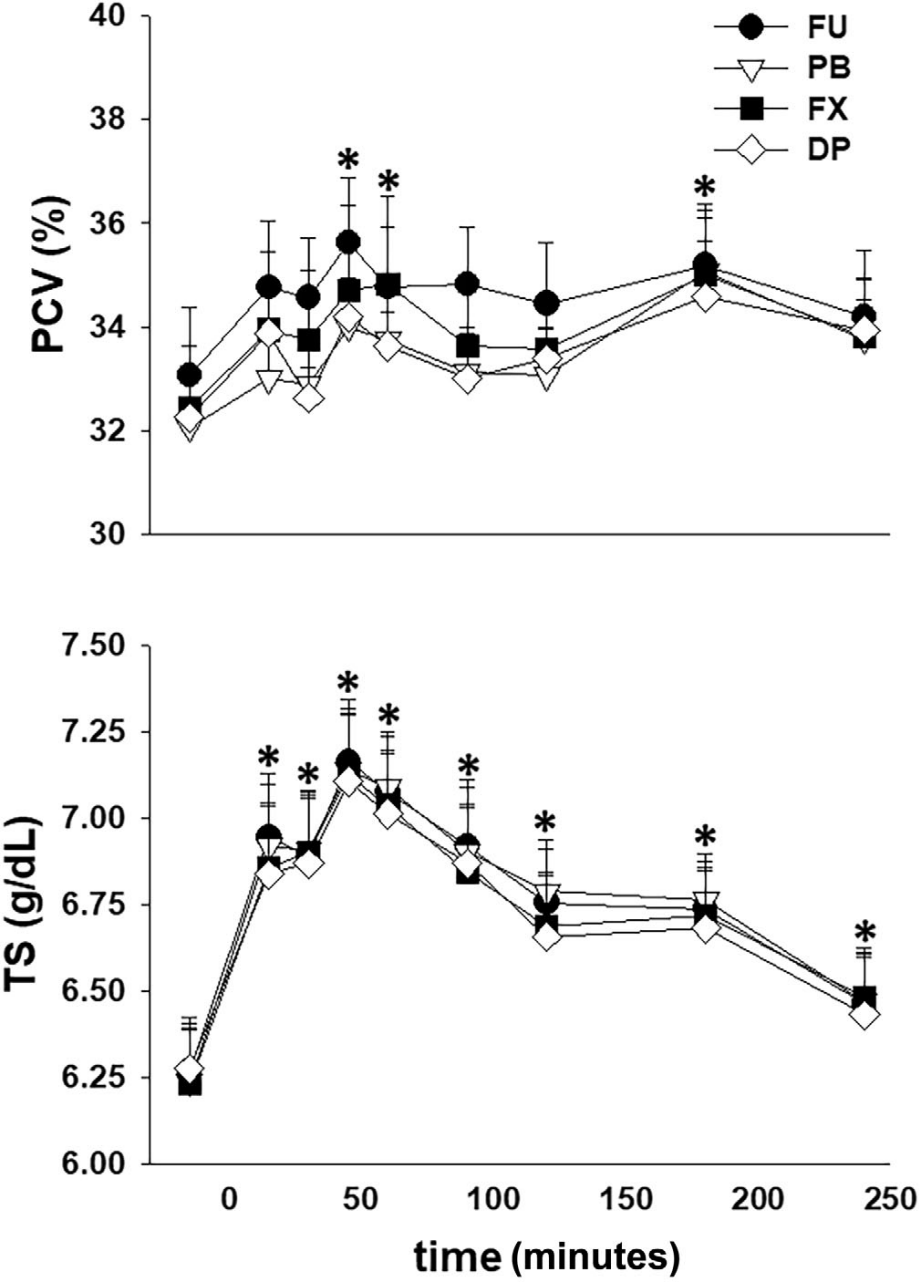
Mean ± SEM values for packed cell volume (PCV) and total solids (TS) in 8 mares before and during 4 hours after furosemide administration (1.0 mg/kg, IV), after pretreatment with 0.9% NaCl (FU, control), phenylbutazone (PB); firocoxib (FX); and dipyrone (DP). * indicates significant difference (*P* < .001) from the −15 minute values over time. There was no significant difference attributable to NSAID treatment.

**FIGURE 2:**
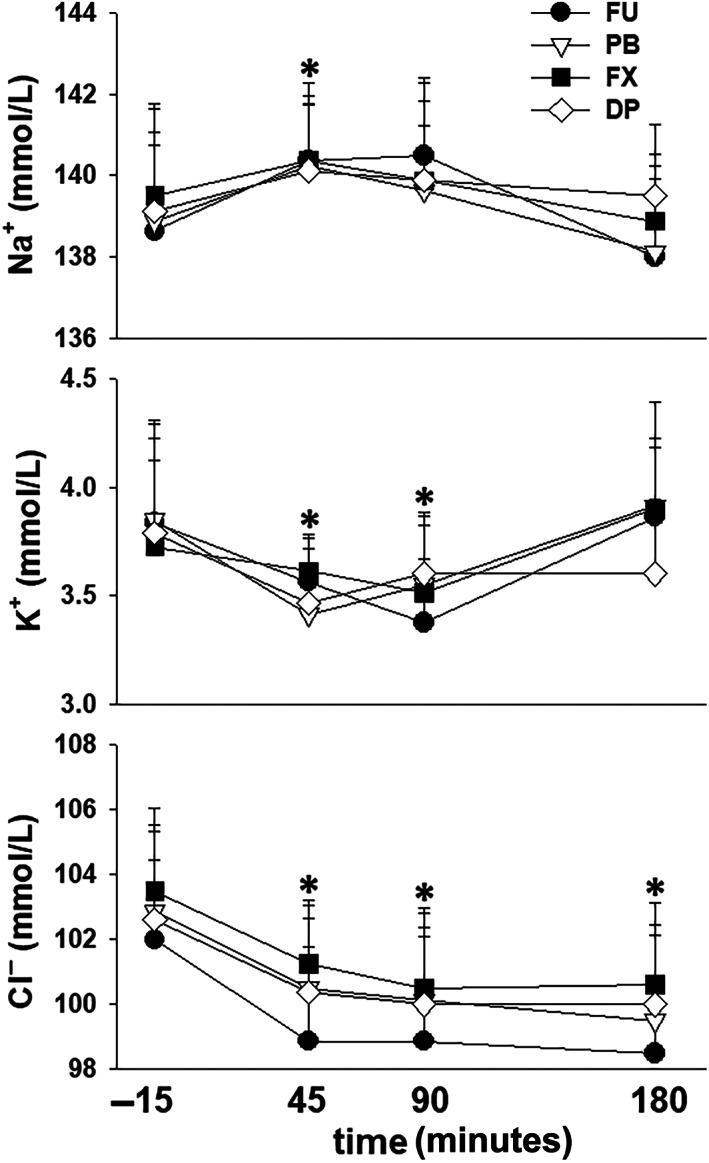
Mean ± SEM values for serum Na^+^, K^+^, and Cl^−^ concentrations in 8 mares before and during 4 hours after furosemide administration (1.0 mg/kg, IV), after pretreatment with 0.9% NaCl (FU, control), phenylbutazone (PB); firocoxib (FX); and dipyrone (DP). * indicates significant difference (*P* < 0.001) from the −15 minute values over time. There was no significant difference attributable to NSAID treatment.

All mares tolerated restraint in stocks and instrumentation without sedation during the experiments. Three of the 32 experiments had to be repeated because of dislodging of a ureteral catheter (n = 2) and leakage of urine around a ureteral catheter into the bladder (n = 1). No changes in HR, either in response to furosemide administration over time (*P* = .24) or between treatments (*P* = .73), were observed. After furosemide administration PCV was increased (*P* < .001) from the −15 minutes value after 45, 60, and 180 minutes and TS was increased (*P* < .001) from the −15 minutes value through 240 minutes. However, there were no differences between treatments (Figure 1). Serum Na^+^ concentration was increased (*P* < .001) from the −15 minutes value at 45 minutes after furosemide administration while serum K^+^ was decreased (*P* < .001) from the −15 minutes value at 45 and 90 minutes after furosemide administration. After furosemide administration serum Cl^−^ was decreased (*P* < .001) from the −15 minutes from 45 through 240 minutes (Figure 2).

**FIGURE 3:**
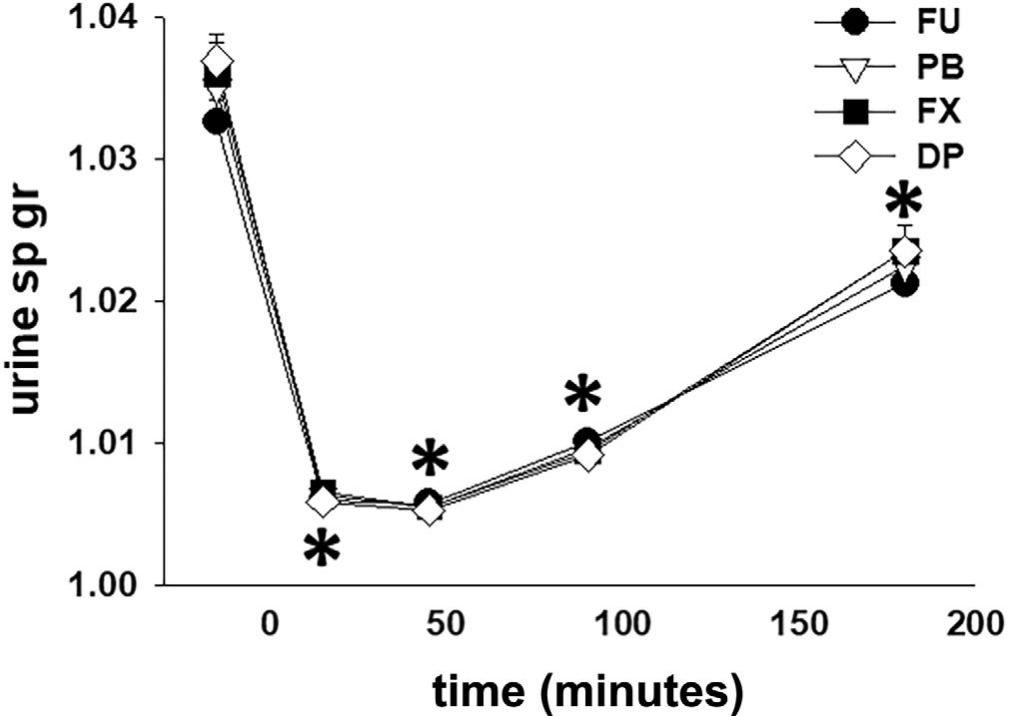
Mean ± SEM values for urine specific gravity (sp gr) in 8 mares before and during 4 hours furosemide administration (1.0 mg/kg, IV), after pretreatment with 0.9% NaCl (FU, control), phenylbutazone (PB); firocoxib (FX); and dipyrone (DP). * indicates significant differences (*P* < .05) from the −30 to 0 minute values.

The total volume of urine produced after furosemide administration was ~25% greater (*P* < .001) with FU, as compared to PB, FX, and DP, and there was no difference in urine volume produced between the 3 NSAID treatments (Table 1). There was no difference (*P* = .27) in total water intake between the treatments: FU 6.4 ± 3.5 L; PB 6.4 ± 4.1 L; FX 7.0 ± 5.5 L; and DP 4.5 ± 4.5 L. With all treatments, USG was decreased (*P* < .001) from the −30 to 0 minute collection period value during all collection periods after furosemide administration and there were no differences in USG between treatment groups (Figure 3).

**TABLE 1:** Mean ± SD urine volume (mL/kg) produced and Na^+^, K^+^, and Cl^−^ excreted (total mmol) by 8 mares during 4 hours after furosemide administration (1.0 mg/kg, IV), after pretreatment with 0.9% NaCl (FU = control), phenylbutazone (PB), firocoxib (FX), or dipyrone (DP).^a^

Treatment	Urine volume (mL/kg)	Urine Cr (mg)	Urine Na^+^ (mmol)	Urine K^+^ (mmol)	Urine Cl^−^ (mmol)
FU	23.4 ± 5.1^A^	2233 ± 528	1169 ± 257^A^	659 ± 187	1702 ± 386^A^
PB	19.1 ± 2.1^B^	2113 ± 658	911 ± 161^B^	593 ± 196	1549 ± 384^B^
FX	17.7 ± 3.5^B^	2236 ± 388	807 ± 146^B^	609 ± 174	1392 ± 248^B^
DP	19.1 ± 3.9^B^	2331 ± 604	916 ± 248^B^	594 ± 160	1500 ± 320^B^

^a^ Different superscript letters in columns indicate a significant difference (*P* < .05).

**FIGURE 4:**
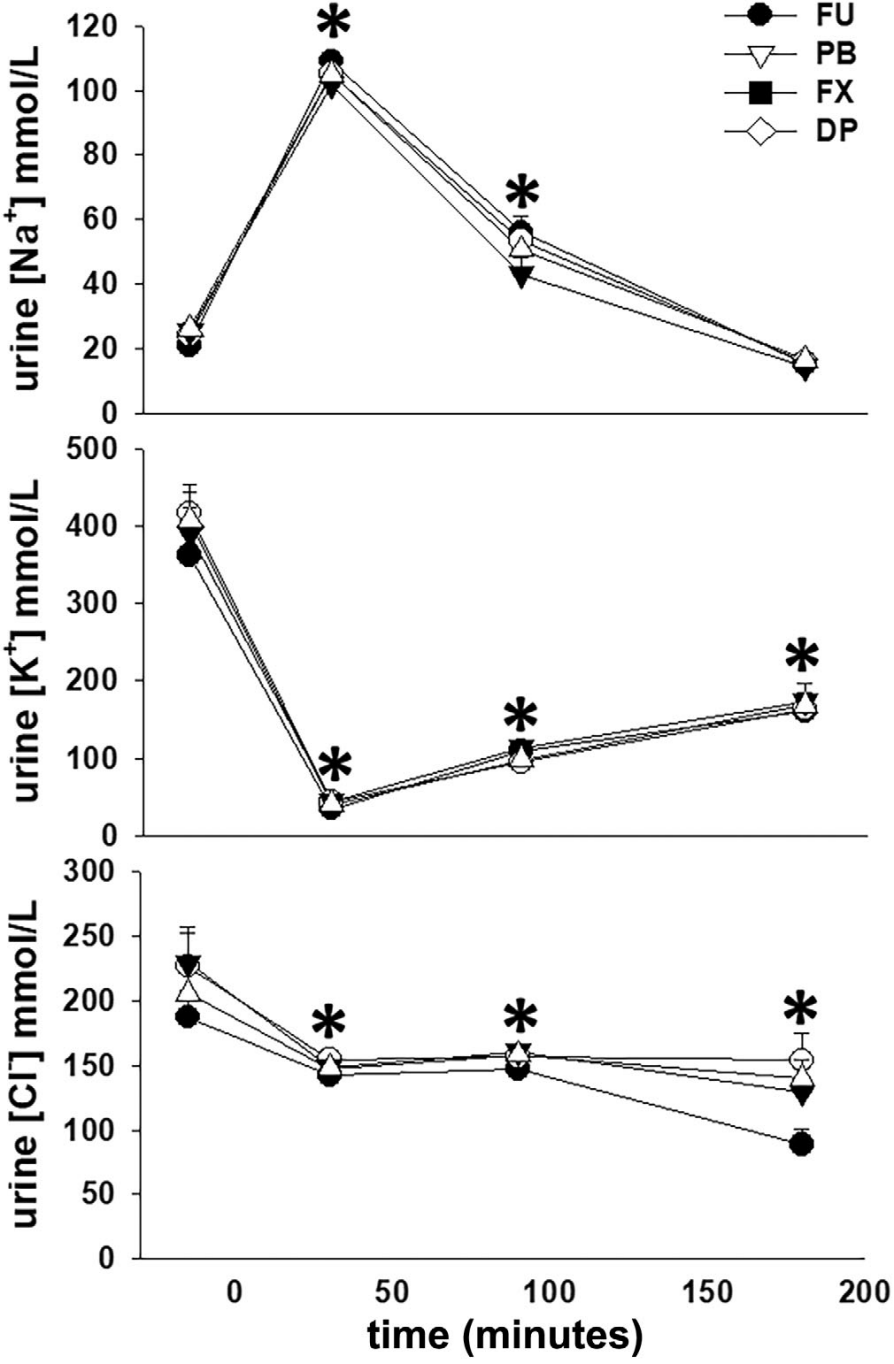
Mean ± SEM values for urine Na^+^, K^+^, and Cl^−^ concentrations in 8 mares before and during 4 hours after furosemide administration (1.0 mg/kg, IV), after pretreatment with 0.9% NaCl (FU, control), phenylbutazone (PB); firocoxib (FX); and dipyrone (DP). * indicates significant differences (*P* < .05) from the −30 to 0 minute values.

Urine Na^+^ concentration was increased (*P* < .001) from the −30 to 0 minute collection period value during the 0 to 60 and 60 to 120 minutes collection periods while urine K^+^ and Cl^−^ concentrations were decreased (P < .001) from the −30 to 0 minute collection period values during all subsequent collection periods after furosemide administration. There were no differences in urine electrolyte concentrations between treatment groups during any collection period (Figure 4). Total excretion of Na^+^ and Cl^−^, not standardized for bodyweight, during the 4 hours after furosemide administration was ~25% and ~13% greater (*P* < .001 for both) with FU, as compared to PB, FX, and DP, and there were no differences between the 3 NSAID treatments (Table 1). Although total excretion of K^+^, again not standardized for bodyweight, during the 4 hours after furosemide administration was ~10% greater for FU, as compared to PB, FX, and DP, this was not a significant finding (*P* = .63) and there were no differences in total K^+^ excretion between the 3 NSAID treatments.

**FIGURE 5:**
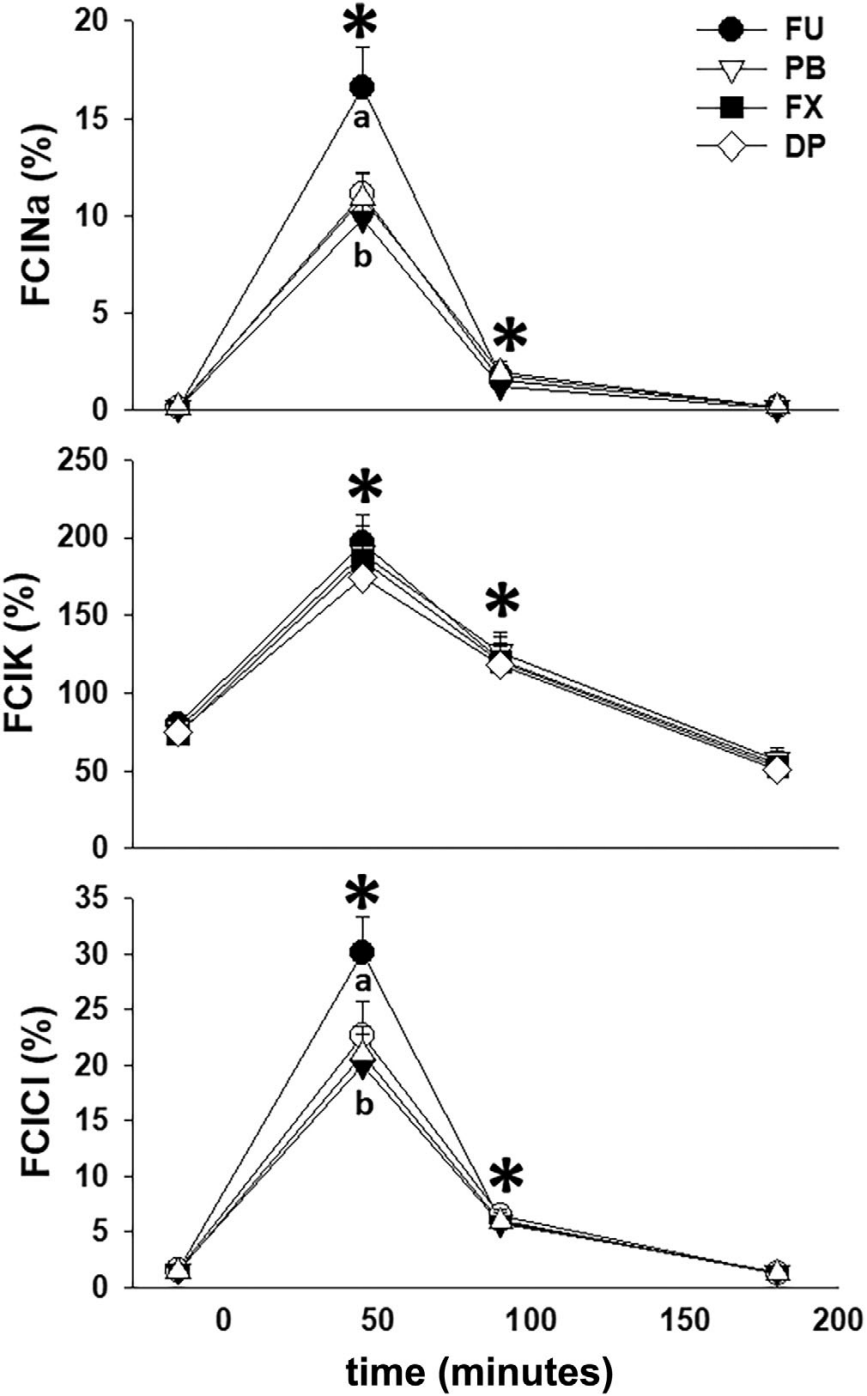
Mean ± SEM values for FCl_Na_, FCl_K_, and FCl_Cl_ in 8 mares before and during 4 hours after furosemide administration (1.0 mg/kg, IV), after pretreatment with 0.9% NaCl (FU, control), phenylbutazone (PB); firocoxib (FX); and dipyrone (DP). * indicates significant differences (*P* < .05) from the −30 to 0 minute values; different letters below lines indicate significant differences between treatments within a urine collection period.

Total Cr excretion during the 4 hours after furosemide administration was not different (*P* = .23) between treatment groups (Table 1). When serum and urine Cr and electrolyte concentrations were used to calculate fractional clearances of Na^+^, K^+^, and Cl^−^ (FCl_Na_, FCl_K_, and FCl_Cl_), all electrolyte clearances were increased (*P* < .01 for all) from −30 to 0 minute collection period values during the 0 to 60 and 60 to 120 minutes collection periods after furosemide administration. Further, increases in FCl_Na_ and FCl_Cl_ during the 0 to 60 minutes collection period were greater (*P* < .01) for FU than the 3 NSAID treatments (Figure 5).

Although mean urine production during the 4 hours after furosemide administration was decreased by a similar degree with all NSAID treatments, there was considerable variation among horses. Specifically, the magnitude of diuresis during the 4 hours after furosemide administration ranged from 16.2 to 30.0 mL/kg for the FU treatment. In comparison to FU, the range of urine volume difference was 72% to 102%, 60% to 91%, and 68% to 111% for PB, FX, and DP, respectively (Figure S1). The differences in urine output between FU and the 3 NSAID treatments were less apparent in horses that had lower values for urine production (<20 mL/kg) with FU during the 4 hours after furosemide administration.

## 4 DISCUSSION

This study compared the effects of a COXIB (FX) and an atypical COX inhibitor (DP) to a nonselective COX inhibitor (PB) on renal function in horses by evaluating the diuretic and natriuretic response to furosemide after NSAID administration. Pretreatment with all NSAIDs resulted in ~25% lower total urine volume after furosemide administration as compared to furosemide alone. Furosemide‐induced natriuresis and chloruresis were similarly attenuated after pretreatment with all NSAIDs. The results refute our hypothesis that pretreatment with FX or DP would have a lesser inhibitory effect on furosemide‐induced diuresis and natriuresis than pretreatment with PB and are consistent with previous studies investigating the renal effects of phenylbutazone and meloxicam in horses.^8^, ^9^, ^10^ We also found considerable, but inconsistent, interindividual variation in both the magnitude and attenuation of furosemide diuresis after treatment with the different NSAIDs.

After furosemide administration, PCV and TS increased in all groups, similar to previous reports.^8^, ^19^, ^20^ The increase in TS was consistent with a 10% to 15% decrease in plasma volume in the hour after furosemide administration.^20^, ^21^ A lesser increase in PCV, as compared to TS, has been attributed to sequestration of erythrocytes within the spleen.^5^, ^19^ Initially, serum Na^+^ concentration increased and serum K^+^ concentration decreased after furosemide administration in all treatment groups; however, these serum electrolyte concentrations returned to prefurosemide values by 180 minutes. We could not find a previous report documenting an increase in serum Na^+^ concentration after furosemide administration to horses. Our horses did not have access to water during movement to stocks and instrumentation (~1.5 hours) and were not offered water until 90 minutes after furosemide administration, despite having free access to hay. Thus, a mild increase in serum Na^+^ concentration could be an expected response to a proportionally greater loss of water, supported by maximal urine Na^+^ concentrations of ~100 mmol/L from 0 to 60 minutes after furosemide administration for all treatments. A decrease in serum K^+^ concentration has been reported after furosemide administration and has been attributed to redistribution of K^+^ to the intracellular space, rather than increased urinary K^+^ excretion.^5^, ^19^, ^21^ However, although urine K^+^ concentration decreased nearly 10‐fold from 0 to 60 minutes after furosemide administration, we also observed a doubling of FCl_K_ during this same collection period suggesting that increased K^+^ excretion could also play a role in development of transient hypokalemia. Finally, serum Cl^−^ concentration decreased 45 minutes after furosemide administration and remained decreased through the remainder of the 4 hours urine collection period. Hypochloremia develops consequent to increased urinary Cl^−^ excretion as evidenced by the minimal decrease in urine Cl^−^ concentration and substantial increase in FCl_Cl_ after furosemide administration.^5^, ^9^, ^19^

In comparison to prefurosemide values, urine Na^+^ concentration and FCl_Na_ increased from 0 to 120 minutes after furosemide administration. In contrast, urine K^+^ and Cl^−^ concentrations decreased and remained lower than baseline values through the remainder of the urine collection period, although FCl_K_ and FCl_Cl_ returned to prefurosemide values from 180 to 240 minutes. Because urine Na^+^ concentration is typically low in horses not receiving dietary Na^+^ supplementation (24 ± 7 mmol/L before furosemide administration in the mares in this study), the nearly 5‐ and 10‐ to 15‐fold increases in urine Na^+^ concentration and FCl_Na_, respectively, were expected responses to furosemide administration with a decrease in Na^+^ reabsorption because of blockade of NKCC‐2 cotransporters in the TAL. In contrast, although urine K^+^ and Cl^−^ concentrations decreased after furosemide administration, baseline values were greater than baseline urine Na^+^ concentration. Consequently, with furosemide‐induced diuresis absolute urine K^+^ and Cl^−^ concentrations decreased (substantially more for K^+^ than Cl^−^) while total excretion and FCl_K_ and FCl_Cl_ increased. All NSAID treatments attenuated the increases in FCl_Na_ and FCl_Cl_ during the initial hour after furosemide administration, consistent with a previous study that investigated the pharmacologic interaction of furosemide and phenylbutazone in horses.^8^ In all, urine electrolyte losses in the 4 hours after furosemide administration (Table 1) were comparable to previous reports^9^, ^19^ and approached ~7 and ~14% of extracellular fluid (ECF) Na^+^ and Cl^−^ contents, respectively, assuming ~14 000 mmol of Na^+^ and ~11 000 mmol of Cl^−^ in the ECF of a 500 kg horse. Despite an increase in FCl_K_ during the 2 hours after furosemide administration, total loss of K^+^ was <3% of intracellular fluid (ICF) K^+^ content (assuming ~28 000 mmol of K^+^ in the ICF of a 500 kg horse).

The primary pharmacologic action of furosemide is attachment to the luminal Cl^−^ binding site of the NKCC‐2 cotransporter in the TAL, leading to decreased solute reabsorption. Because 20% and 25% of Na^+^ and Cl^−^ are reabsorbed via the NKCC‐2 cotransporter in this nephron segment, furosemide administration results in a large increase in solute delivery to the distal nephron and increased output of urine.^5^, ^6^, ^22^ However, furosemide administration also leads to changes in systemic and renal hemodynamics, with a net effect of increasing RBF, and potentially GFR.^5^, ^6^ Further, PGE_2_ production in TAL cells increases in response to furosemide administration through combined effects of increased release of arachidonic acid from phospholipids, increased COX‐2 expression, and decreased degradation of PGE_2_ by renal PGE_2_ 9‐ketoreducatse and 15‐hydroxyprostaglandin dehydrogenase.^7^, ^23^, ^24^ Local action of PGE_2_ in TAL and MD cells contributes to a further decrease in solute reabsorption. Finally, furosemide‐induced PGE_2_ production in cortical collecting duct epithelial cells can antagonize the antidiuretic effect of AVP.^4^, ^25^

There are several mechanisms by which administration of NSAIDS could attenuate the diuretic and saliuretic responses to furosemide. First, pretreatment with phenylbutazone abolished furosemide‐induced changes in systemic hemodynamics, including decreases in stroke volume and cardiac output and right atrial and right ventricular pressures, in resting horses.^8^ However, inhibition of these hemodynamic responses to furosemide administration was considered unlikely to contribute substantially to attenuation of furosemide‐induced diuresis.^5^, ^8^ Next, because furosemide administration directly stimulates PGE_2_ production in TAL and MD cells, further decreasing solute absorption, inhibition of COX‐2 derived PGE_2_ in this nephron segment could attenuate inhibition of solute reabsorption and diuresis.^23^ Finally, production of either a concentrated or dilute final urine is fine‐tuned by AVP action to regulate insertion of aquaporins (water channels) in cortical and medullary collecting duct epithelial cells. Attenuation of AVP‐induced water reabsorption across isolated perfused rabbit collecting ducts by PGE_1_ was first documented in 1968 (aquaporins were not discovered until 1992).^4^ It was proposed that PGs dampened the activity of AVP to prevent overshoots in AVP‐induced collecting duct permeability.^26^ Fifty years later the actions of PGE_1_ and PGE_2_ are now recognized to be mediated through binding to 1 of 4 subtype receptors (EP1‐EP4) with incongruous effects on collecting duct water permeabilty.^4^ PGE_2_ action on EP4 and EP2 receptors increase intracellular cAMP, promoting phosphorylation, translocation, and expression of aquaporin 2 (AQP2), thereby increasing collecting duct water permeability and urine concentration. In contrast, PGE_2_ action on EP1 and EP3 receptors appears to downregulate AQP2 function by increasing intracellular Ca^++^ and attenuating cAMP production, respectively, leading to diuresis.^4^, ^27^ Currently, this complex interaction of PGE_2_ with its multiple receptors is incompletely understood but in studies examining the effects of NSAIDs on furosemide‐induced diuresis, NSAIDs attenuate diuresis by up to 50%.^3^, ^4^, ^6^

Our study was not designed to determine the mechanism(s) by which NSAIDs attenuate furosemide‐induced diuresis; thus, the proportion of saliuresis attributable to decreased solute reabsorption versus potential interference with AVP action on collecting ducts remains uncertain. Our interest was in comparing the effects of a nonspecific COX inhibitor (phenylbutazone), a COXIB (firocoxib), and an atypical NSAID (dipyrone) on the diuretic responses of horses to furosemide administration. Again, we found no difference in attenuation of furosemide‐induced diuresis and saliuresis after pretreatment with these 3 NSAIDs. A limitation of our investigation was that we studied healthy, euhydrated mares and it is possible that these NSAIDs could have different effects in dehydrated or diseased horses with altered RBF. Acknowledging this limitation, our data do suggest that use of COXIBs might not be “renoprotective” in diseased equids, as compared to nonselective COX inhibitors. Additionally, the variability in furosemide‐induced diuresis observed between individual mares in this study, with and without pretreatment with NSAIDs, was considerable and emphasizes the need to monitor renal function when using all NSAIDs in diseased equids.

## CONFLICT OF INTEREST DECLARATION

Authors declare no conflict of interest.

## OFF‐LABEL ANTIMICROBIAL DECLARATION

Trimethoprim‐sulfamethoxazole used in the horses included in this manuscript is not labeled for prophylaxis or treatment of urinary tract infection or similar indications in the United States.

## INSTITUTIONAL ANIMAL CARE AND USE COMMITTEE (IACUC) OR OTHER APPROVAL DECLARATION

Approved by the Michigan State University IACUC (PROTO202000077).

## HUMAN ETHICS APPROVAL DECLARATION

Authors declare human ethics approval was not needed for this study.

## Supporting information


**Data S1.** Supplementary information.
