# Supplementary material for: Effects of phenylbutazone, firocoxib, and dipyrone on the diuretic response to furosemide in horses
Source: J Vet Intern Med. 2023 Oct 26;37(6):2544–51. doi: 10.1111/jvim.16914 (PMC10658499; doi:10.1111/jvim.16914)
Supplement: Supplementary file 1 — Data S1. Supplementary information. [file JVIM-37-2544-s001.pdf]

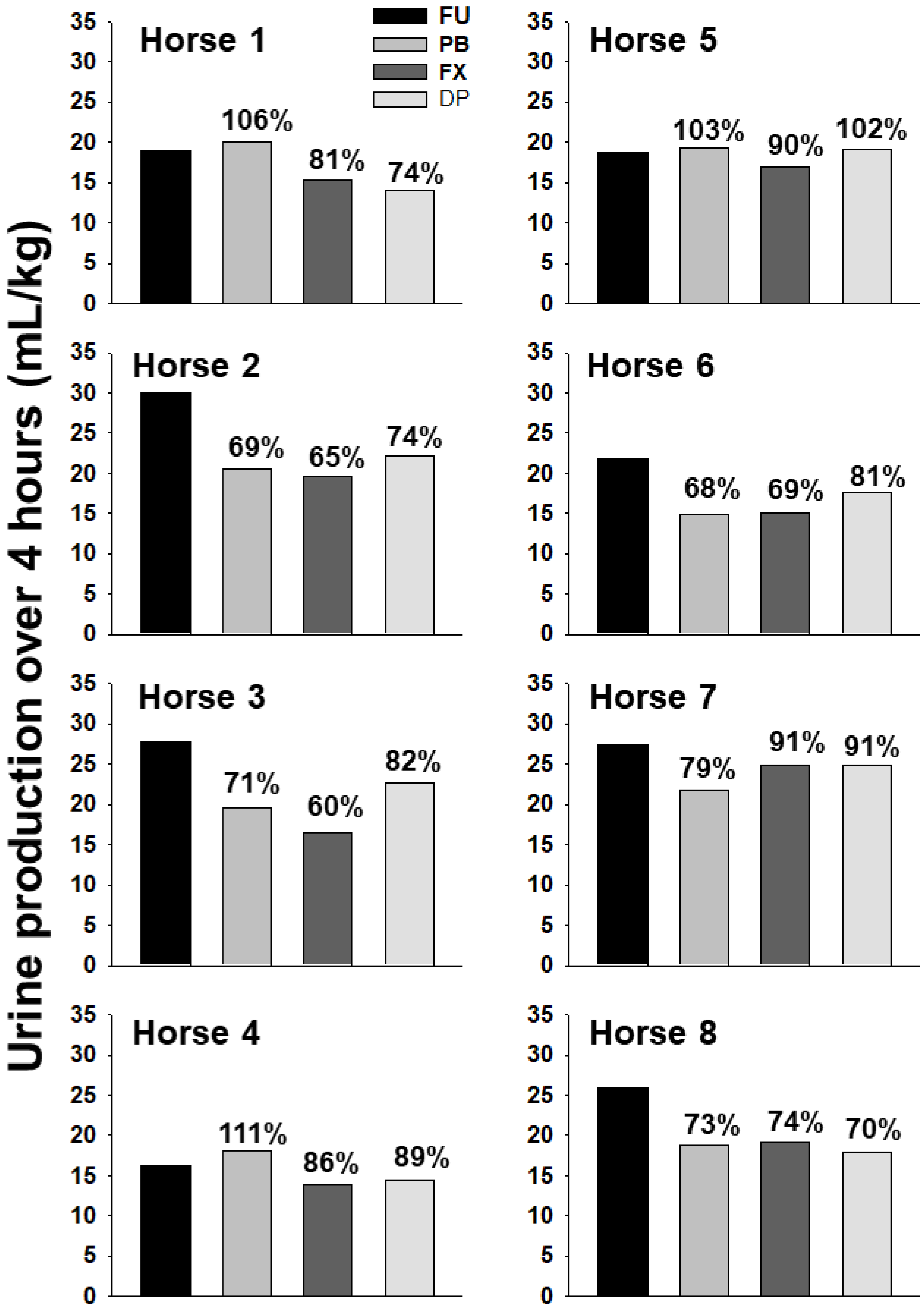

Urine production over 4 hours (mL/kg)
FU
PB
FX
DP
Horse 1
106%
81%
74%
Horse 2
69%
65%
74%
Horse 3
71%
60%
82%
Horse 4
111%
86%
89%
Horse 5
103%
90%
102%
Horse 6
68%
69%
81%
Horse 7
79%
91%
91%
Horse 8
73%
74%
70%
0
5
10
15
20
25
30
35

Supplementary Figure 1. Individual values for urine volume (mL/kg) produced in eight mares in the 4 h after furosemide administration (1.0 mg/kg, IV), following pretreatment with 0.9% NaCl (FU, control), phenylbutazone (PB); firocoxib (FX; or dipyrone (DP). Percentage values above each bar are for comparison to the urine volume after the control (FU) treatment.
